# Irrigation of Abdomen With Imipenem Solution Decreases Surgical Site Infections in Patients With Perforated Appendicitis: A Randomized Clinical Trial

**DOI:** 10.5812/ircmj.12732

**Published:** 2014-04-05

**Authors:** Mohammad Ali Hesami, Hamid Alipour, Hamed Nikoupour Daylami, Bijan Alipour, Shahrzad Bazargan-Hejazi, Alireza Ahmadi

**Affiliations:** 1 Department of Surgery, Imam Reza Hospital, Kermanshah University of Medical Sciences, Kermanshah, IR Iran; 2 Research Center for Immunodeficiencies, Children’s Medical Center, Tehran University of Medical Sciences, Tehran, IR Iran; 3 Department of Psychiatry, Charles R. Drew University of Medicine and Sciences, and David Geffen School of Medicine, UCLA, USA; 4 Department of Public Health Sciences, Division of Social Medicine, Karolinska Institute, Stockholm, Sweden; 5 Department of Anesthesiology, Imam Reza Hospital, Kermanshah University of Medical Sciences, Kermanshah, IR Iran

**Keywords:** Appendicitis, Peritoneal Lavage, Surgical Site Infection, Wound Infection, Abdominal Abscess, Iran

## Abstract

**Background:**

Perforated appendicitis is one of the most common causes of acute abdomen requiring emergent surgery for immediate appendectomy and peritoneal cavity irrigation; however, the efficacy of irrigation with antibiotic solutions is controversial.

**Objectives:**

The aim of this study was to assess the efficacy of imipenem solution irrigation on post-operative surgical site infections (SSIs), hospital length of stay, and hospital costs. We hypothesized that there would be lower rate of SSIs, a shorter hospital stay, and lower hospital cost in patients with perforated appendicitis who received peritoneal cavity irrigation with imipenem solution in comparison to their counterparts who received irrigation with normal saline.

**Patients and Methods:**

In this randomized single-blind parallel-group clinical trial, we enrolled 90 patients with perforated appendicitis with 12-50 years of age and randomly allocated them into experimental group (n = 45) and control group (n = 45). The control group received peritoneal irrigation with normal saline (0.9%) and experimental group underwent peritoneal irrigation with imipenem solution (1 mg/mL). All surgical procedures were performed in Imam Reza Hospital of Kermanshah University of Medical Sciences. The study primary outcome was surgical site infections (including wound infection and abdominal abscess) and the secondary outcomes were length of hospital stay and hospital cost. Chi-squared and t-tests were used to analyze the study data.

**Results:**

Imipenem solution irrigation was associated with significant clinical improvement at one-month follow-up. The experimental group presented with significantly lower rate of SSIs and shorter length of hospital stay. The experimental group had lower rate of SSIs compared to the control group (4.4% vs. 22.2%, respectively) (p= 0.013). The duration of hospital stay was nearly one day longer in control group (5.84 ± 2.58 days) vs. experimental group (4.91 ± 1.29 days) (P = 0.034), and hospital costs were $50 lower in experimental group ($500 ± $292) vs. control group ($450 ± $170) (P = 0.281).

**Conclusions:**

The study findings revealed that peritoneal lavage with imipenem solution (1 mg/mL) decreases the rate of post-operative SSIs in patients with perforated appendicitis in comparison to patients irrigated with normal saline alone. These patients also had shorter hospital stay, and lower hospital costs.

## 1. Background

Appendicitis is one of the most common abdominal conditions requiring surgery. In some cases if the duration of the disease prolongs and/or timely treatment is not received, perforation might occur. The overall rate of perforated appendicitis is 25.8% (1, 2). The incidence of major complications after appendectomy is associated with appendiceal rupture. The most important complication is surgical site infections (SSIs), which include wound infection and intra-abdominal abscess. SSIs are significantly higher in perforated appendicitis rather than non-perforated ones (17% and 6%, respectively) (3).

Although all surgeons agree that the treatment method for perforated appendicitis is immediate appendectomy and irrigation of the peritoneal cavity, specific details of the pre- and intraoperative management of these patients such as antibiotic irrigation of the peritoneal cavity are frequently debated (3). In the past 50 years, numerous antibiotics and antiseptic solutions like ampicillin, metronidazole, doxycycline, cefazolin, cephotetan, cephalothin, cephaloridine, bacitracin, lincomycin, gentamicin, kanamycin, and Dakin’s solution have been used as irrigation solution for the treatment of peritonitis; however, there are still controversies about their effectiveness (4-9). There is a general agreement that antibiotic lavage is safe, but there has been little evidence to support its efficacy compared to the control group (10-15).

The results of recent experimental studies and meta-analysis reveal that the use of antibiotic lavage is associated with a more favorable treatment outcome compared to irrigation with normal saline (16, 17). In a more recent study, Clindamycin-Gentamicin combination lavage was shown to be effective at preventing surgical-site infections of cancerous patients who underwent colorectal resection (18). Another recent retrospective study found that peritoneal irrigation with imipenem solution (1 mg/mL) was more beneficial, compared to irrigation with normal saline, in decreasing the risk of post-operative SSIs (8). This finding suggests that imipenem may be a good choice as a peritoneal washing solution, because it is a wide spectrum antibiotic with highly bactericidal activity on microorganisms causing peritonitis, including facultative gram-negative enteric bacteria and obligate anaerobe rods. There is a paucity of prospective research regarding whether abdominal cavity irrigation with imipenem solution would decrease the rate of post-operative surgical site infections.

## 2. Objectives

The aim of this randomized clinical trial was to evaluate the efficacy of peritoneal cavity irrigation with imipenem solution in patients with perforated appendicitis concerning post-operative SSIs (including wound infection and abdominal abscess), length of hospital stay, and hospital costs. With regard to the findings from the previous studies, we hypothesized that patients with perforated appendicitis who received peritoneal cavity irrigation, compared to their counterparts who received irrigation with normal saline, would have a lower rate of SSIs, a shorter hospital stay, and a lower hospital costs.

## 3. Patients and Methods

### 3.1. Design, Recruitment and Study Sample

**Figure 1.**
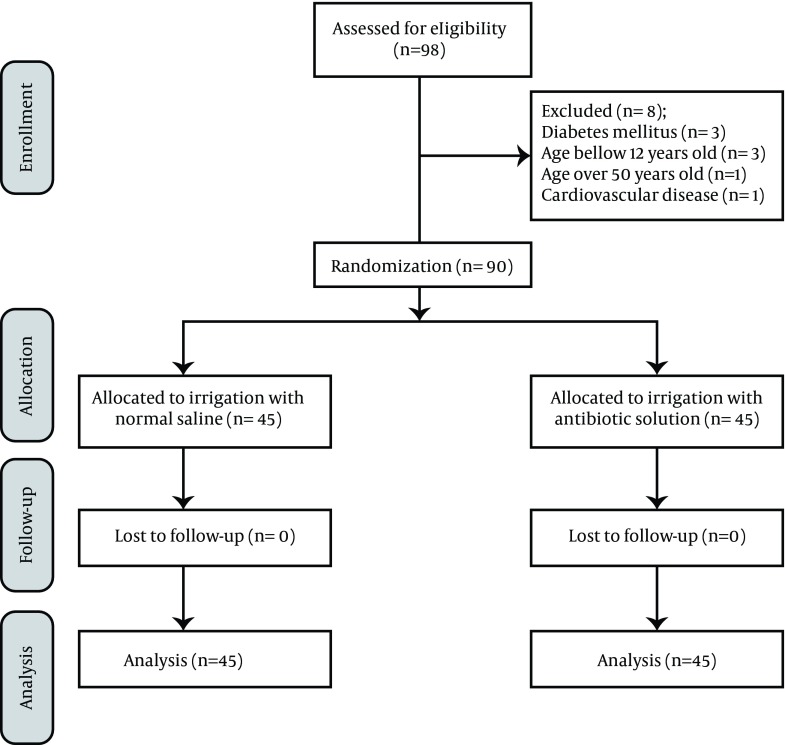
CONSORT Flow Diagram of Recruitment Through Final Analysis

This was a single-blind parallel-group randomized clinical trial study. All patients aged between 12- to 50-year-old who were emergently operated due to acute abdomen between April 2010 and January 2011 were enrolled in the study. Patients with immunodeficiency, co-morbidities such as diabetes, cardiovascular, renal, pulmonary, or hepatic diseases, and those younger than 12 or older than 50 years of age (since there are lower rates of SSIs in children and higher rates in elderly) were excluded from the study. Prior to the surgery, eligible patients and/or their caregivers were informed about the study and its voluntary participation. Subsequently, informed consent was obtained to enroll patients in the study if intra-operative findings (e.g. a hole in appendix, abscess or puss within the abdomen) had confirmed the diagnosis of perforated appendicitis as the cause of peritonitis (Figure 1). The study was conducted at Imam Reza hospital in Kermanshah, an affiliated hospital of Kermanshah University of Medical Sciences (KUMS), Iran.

### 3.2. Study Variables

Data were collected on patient socio-demographic characteristics including age and sex as well as baseline white blood cell count and body temperature. These data were collected from the patient’s chart. Primary outcome variables were post-operative complications, i.e. SSIs including wound infection and abdominal abscess. Wound infection was confirmed by observing purulent discharge, redness, inflammation, and the need to reopen a wound for its management. The presence of intra-abdominal abscess was assessed by the surgeon examining patients with abdominal pain, fullness, and fever, and was confirmed with sonography. The study secondary outcome variables were length of hospital stay and hospital costs. Hospital costs were calculated based on the government estimates of medical expenses in Iran.

### 3.3. Sample Size, Randomization and Ethical Approval

The findings of previous descriptive studies showed that the rates of SSIs in patients with perforated appendicitis who had received irrigation with normal saline and imipenem solution were 19.6% and 1.5%, respectively (8). Assuming 5% significance and 90% power, the sample size required to ensure high power in each study group was 45. Patients were randomly allocated into the two study groups using computer generated random numbers. The study protocol was approved by the ethical committee of Kermanshah University of Medical Sciences. (7/420/12992/P, Jun 14, 2010).

### 3.4. Intervention

All patients received intravenous antibiotic (ampicillin-sulbactam 3 g) 30 minutes before the surgery. The patients were randomly allocated, by persons external to the study, into two treatment groups with allocation ratio of 1:1. In the first group (control group), patients' abdomen was irrigated with normal saline and in the second group (experimental group) with normal saline plus imipenem (1 mg/mL). In all patients, the fascia was closed with vicryl sutures. The skin and subcutaneous tissues were left open for delayed primary closure. In both groups, wounds were irrigated with normal saline twice a day and were closed four days later. During this period, all patients received systemic antibiotic (ampicillin-sulbactam 3 g qid). Patients’ follow-up evaluations for possible SSIs took place in an outpatient clinic setting by surgery resident, once a week up to one month. The patients and all medical intervention staff including radiologists, except the surgeons, were blinded to the treatment allocation.

### 3.5. Data Analysis

The data for this study were analyzed in two sections; first, we used descriptive statistics such as means, rates, and standard deviation to present pattern of distributions of the main variables in the study groups. Chi-squared tests were used for comparing numbers of SSIs in groups, and assuming the violation of parametric assumption, we used t-test to compare means lengths of hospital stay and hospital costs between the two study groups. There is ample evidence indicating that power of t-test and F-test under violation of the assumption of homogeneity of variance are comparable, i.e. they are hardly affected by non-normality of the sample distribution (19). The level of p to reject the null hypothesis was set at ≤ 0.05. The data were analyzed using SPSS version 17 (SPSS Inc, Chicago, Illinois, USA).

## 4. Results

Of the initial 98 patients who were approached as potential participants in this study, 90 patients with acute abdomen due to perforated appendicitis were enrolled and eight patients were excluded (See Figure 1 for details). Half of the enrolled patients underwent peritoneal irrigation with normal saline (control group) and the other half were irrigated by imipenem solution (1 mg/ml) (experimental group). Patients in the experimental group were mostly male (60%). The mean age of these patients was 29.4 ± 12.2 years. Of the patients in the control group, 57.7% were males and the mean age of this group was 24.5 ± 11.5 years. There was no statistically significant difference with regard to age, gender, white blood cell count, and temperature between control and experimental groups (Table 1). Of the 90 patients in the study, 12 (13.3%) had post-operative infection complications; ten in control group and two in the experimental group (P = 0.013) (Table 2). Of these postoperative complication cases, seven patients had wound infections, five patients in control and two patients in experimental group (P = 0.23); seven patients had abdominal abscess, six patients in control and one patient in experimental group (P = 0.049). As indicated in Table 3, the averages lengths of hospital stay and costs for all patients were 5.4 days and $475, respectively. The minimum and maximum duration of hospital stay were four and 15 days, respectively. In regards to the hospital costs, the minimum and maximum costs were $300 and $1600, respectively. Mean length of hospital stay was nearly one day more and significantly longer in control group compared to the experimental group (5.84 ± 2.58 and 4.9 ± 1.29 days, respectively; P = 0.034). The average hospital cost in the control group was $500 while it was $450 in the experimental group (P = 0.28), even though patients in the experimental group paid $50 more than controls for administration of imipenem. Table 4 indicates that the mean length of stay in patients with SSIs was nearly five days longer than the patients without SSIs and their hospital costs were more than two times ($550) of the patients without SSIs.

**Table 1.** Basic Characteristics of the Study Groups at the Baseline (n = 45) ^a^

	Control	Experimental
**Age, y**	24.5 (11.5)	29.4 (12.2)
**Gender**		
Male	57.7	60
Female	42.2	40
**WBC count**	15.6 (4.8)	14.9 (5)
**Temperature**	38 (0.7)	37.9 (0.9)

^a^ Data are presented in NO. (%).

**Table 2.** The Rate of Postoperative Complication in Control and Experimental groups (n = 45) ^a^

	Control	Experimental	P Value
**Patients with surgical site infection**	10 (22.2)	2 (4.4)	0.013
**Patients with wound infections**	5 (11.1)	2 (4.4)	0.23
**Patients with abdominal abscess**	6 (13.3)	1 (2.2)	0.049

^a^ Data are presented in NO. (%).

**Table 3.** Distribution of the Length of Hospital Stay and Cost in the Sample ^a^

	Length of Hospital Stay, d	Hospital Cost
**Total sample (n = 90)**	5.4 ± 2.09	$475 ± $240
**Experimental (n = 45)**	5.84 ± 2.58	$500 ± $292
**Control (n = 45)**	4.9 ± 1.29	$450 ± $170
**P value**	0.034	0.281

^a^ Data are presented in Mean ± SD.

**Table 4.** The Difference in Length of Hospital Stay and Charges of Patients With Surgical Site infections and Without It ^a^

	Patients With SSIs	Patients Without Surgical Site infections	P Value
**Mean length of hospital stay (Days)**	9.58 ± 2.53	4.73 ± 0.96	P < 0.0001
**Hospital charge**	$960 ± $382	$400 ± $55	P < 0.0001

^a^ Data are presented in Mean ± SD.

## 5. Discussion

The use of antibiotic irrigation of the peritoneal cavity in perforated appendicitis is controversial (3). Our findings demonstrated that patients who had received irrigation with imipenem solution had significantly fewer SSIs compared to their counterparts who had received irrigation with normal saline (4.4% vs. 22.2%). Moreover, mean length of hospital stay and mean hospital costs were lowered in the treatment group. Since the introduction of antimicrobial to general surgery in the 1940s, there has been an interest in their topical application in peritonitis (20). Parcells et al. published a retrospective study of patients with appendicitis (perforated and not perforated) where they used three types of peritoneal irrigations: saline, Dakin’s solution, and imipenem (8). They reported that the rate of SSIs were significantly lower in imipenem group compared to the normal saline group (1.5% vs. 19.6%). As they indicated in their discussion, there were few weaknesses in their study, which the most important using retrospective design.

Similar to Parcells et al., we used imipenem (1 mg/mL) as an irrigation solution based on its effectiveness against gram-negative enteric bacteria or obligate anaerobic bacilli, the causative pathogens in perforated appendicitis. We also administered same parenteral antibiotic (i.e. ampicillin-sulbactam 3 g) in both study groups. However, we improved the weakness of their study by designing a single-blind randomized clinical trial. In our study, there were significantly fewer SSIs in the patients irrigated with imipenem than those irrigated with normal saline (P = 0.013). This suggested high efficacy and good coverage of imipenem against colonic bacteria.

Rambo et al. in a prospective and double-blind study found that copious cephalothin irrigation was not better or worse than copious saline irrigation in patients with peritonitis (21). Cephalothin is a first generation cephalosporin that mostly covers gram-positive bacteria like staphylococci and streptococci. Cephalothin does not have a good coverage on enteric bacteria, which are mostly facultative gram-negatives and obligate anaerobe bacilli. Despite an insufficient antimicrobial coverage, there were fewer wound infections, abdominal abscesses, and deaths in the intervention group compared to the control group although the differences were not statistically significant. This Lack of significant finding in Rambo’s study could be due to inclusion of different causes of peritonitis in their study such as colon perforation, bladder perforation, peptic perforation, gangrenous bowel, appendicitis, pancreatitis, and tubo-ovarian abscess. Different causes of peritonitis with different bacteriologic precedence might need administration of different and specific antibiotics. In the current study, we only included patients with similar bacteriologic combination as a result of perforated appendicitis. This might explain the favorable outcome in our study. Further prospective studies are needed to replicate our findings, as well as the efficacy of peritoneal irrigation in producing favorable outcome for different causes of peritonitis. Previous findings suggest that the shorter interval between peritonitis and initiation of peritoneal cavity irrigation by an antibiotic solution was associated with better outcome. For example, in a meta-analysis of 23 experimental studies on peritonitis in animals, the efficacies of various antibiotics including imipenem were analyzed (17). The authors concluded that the death rate was significantly higher in groups underwent saline irrigation compared to groups that was treated with antibiotic lavage. They suggested that the one to two hours interval between peritonitis and initiation of irrigation might have been the determining factor in obtaining better outcome in these studies. Ruiz-Tovar et al. (18) studied colorectal cancer patients who underwent elective curative surgery. They reported that the antibiotic solution irrigation, a combination of gentamicin (240 mg) and clindamycin (600 mg) in 500 mL of saline, was effective in decreasing the rate of SSIs. As noted in the current study, this combination also sufficiently covers causative-colonic bacteria. An alternative explanation for the favorable outcome in their study could be that the antibiotic solution irrigation was administered within one hour of peritoneal cavity contamination during the surgery.

In our study, despite the emergency condition of the patients, imipenem was effective in decreasing the rate of postoperative SSIs in patients with perforated appendicitis. This is an important finding since in emergency settings there are longer intervals between leak of bacteria to peritoneal cavity and initiation of peritoneal cavity irrigation; therefore, bacterial load is higher when surgery starts. This might explain why there were fewer peritonitis studies in humans with favorable outcomes compared to experimental peritonitis studies in animals. Larger retrospective studies are needed to assess the efficacy of imipenem in peritonitis patients and controlling for the interval between leak of bacteria to peritoneal cavity and initiation of operation. While patients in the experimental group paid $50 more than the controls for administration of imipenem, the mean hospital costs for the experimental group was $50 less than that of the control group. This could have been due to the fact that the mean length of hospital stay was a day longer in control group or the extra costs that patients with SSIs were charged for reoperation or percutaneous drainage. Indeed, patients with abdominal abscess who received reoperation had the longest duration of stay and the highest hospital costs in our study. These costs were calculated based on public hospital charges in Iran. A saving of $50 hospital cost is almost a 10% reduction in average costs of patients with perforated appendicitis. Our findings suggested that it might be due to lower rate of post-operative complications by irrigation of peritoneal cavity using imipenem solution.

Our study has a few limitations. According to some studies, antibiotic irrigation might induce adhesion band formation (17, 22). Although no adhesion band formation was detected in patients irrigated with imipenem solution during our study period, a longer follow-up of these patients could have helped with better assessment of the intra-abdominal adhesion band formation. Another limitation of our study was our inability to assess/predict antibiotic resistance. Imipenem as a high potency antibiotic might cause colonization of resistant bacteria. In Parcells et al. study no antibiotic resistance was detected after ten years of its initiation, and imipenem remained as one of the most effective antibiotics against gram-negative infections (8). In our study, the number of patients who received irrigation with imipenem was limited (n = 45), and we used shorter follow-up time. There is a need for further clinical trials to determine whether bacterial antibiotic resistance should be considered as an important side effect of this treatment. Despite the limitations to our study, this is amongst a few existing studies that have used imipenem in emergency clinical trial setting and concluded that it would reduce surgical site infections rate.

The use of imipenem solution (1 mg/mL) for peritoneal cavity irrigation in patients with perforated appendicitis compared to the irrigation with normal saline reduced the rate of postoperative SSIs, shortened the length of hospital stay, and subsequently reduced the overall hospital costs. Further double-blind clinical trials with larger sample and longer follow-up time are needed to determine whether administration of imipenem solution leads to formation of resistant group bacteria or would causes any other adverse effect.

## References

[1] Flum DR, Koepsell T (2002). The clinical and economic correlates of misdiagnosed appendicitis: nationwide analysis.. Arch Surg..

[2] Burkitt DP (1971). The aetiology of appendicitis.. Br J Surg..

[3] Jaffe BM, Berger DH, Brunicardi FC (2010). The appendix.. Schwartz's principles of surgery..

[4] Hunt JA, Rivlin ME, Clarebout HJ (1975). Antibiotic peritoneal lavage in severe peritonitis. A preliminary assessment.. S Afr Med J..

[5] Stewart DJ, Matheson NA (1978). Peritoneal lavage in appendicular peritonitis.. Br J Surg..

[6] Beatty GL, Mincks JR, Pulaski EJ (1956). Acute appendicitis, tetracycline prophylaxis and wound infection.. Antibiotic Med Clin Ther..

[7] Longland CJ, Gray JG, Lees W, Garrett JA (1971). The prevention of infection in appendicectomy wounds.. Br J Surg..

[8] Parcells JP, Mileski JP, Gnagy FT, Haragan AF, Mileski WJ (2009). Using antimicrobial solution for irrigation in appendicitis to lower surgical site infection rates.. Am J Surg..

[9] Platell C, Papadimitriou JM, Hall JC (2000). The influence of lavage on peritonitis.. J Am Coll Surg..

[10] Burnett WE, Brown GR, Jr., Rosemond GP, Caswell HT, Buchor RB, Tyson RR (1957). The treatment of peritonitis using peritoneal lavage.. Ann Surg..

[11] Saha SK (1996). Efficacy of metronidazole lavage in treatment of intraperitoneal sepsis. A prospective study.. Dig Dis Sci..

[12] Moukhtar M, Romney S (1980). Continuous intraperitoneal antibiotic lavage in the management of purulent sepsis of the pelvis.. Surg Gynecol Obstet..

[13] Stephen M, Loewenthal J (1979). Continuing peritoneal lavage in high-risk peritonitis.. Surgery..

[14] Atkins RC, Scott DF, Holdsworth SR, Davidson AJ (1976). Prolonged antibiotic peritoneal lavage in the management of gross generalized peritonitis.. Med J Aust..

[15] Haffner JF, Eng J, Lotveit T, Aune S (1976). Peritoneal lavage with doxycycline in acute diffuse peritonitis.. Ann Chir Gynaecol Suppl..

[16] Jallouli M, Hakim A, Znazen A, Sahnoun Z, Kallel H, Zghal K (2009). Rifamycin lavage in the treatment of experimental intra-abdominal infection.. J Surg Res..

[17] Qadan M, Dajani D, Dickinson A, Polk HC (2010). Meta-analysis of the effect of peritoneal lavage on survival in experimental peritonitis.. Br J Surg..

[18] Ruiz-Tovar J, Santos J, Arroyo A, Llavero C, Armananzas L, Lopez-Delgado A (2012). Effect of peritoneal lavage with clindamycin-gentamicin solution on infections after elective colorectal cancer surgery.. J Am Coll Surg..

[19] Zimmerman DW (2004). A note on preliminary tests of equality of variances.. Br J Math Stat Psychol..

[20] Perdue PW, Kazarian KK, Nevola J, Law WR, Williams T (1994). The use of local and systemic antibiotics in rat fecal peritonitis.. J Surg Res..

[21] Rambo WM (1972). Irrigation of the peritoneal cavity with cephalothin.. Am J Surg..

[22] Rappaport WD, Holcomb M, Valente J, Chvapil M (1989). Antibiotic irrigation and the formation of intraabdominal adhesions.. Am J Surg..

